# Increased expression of fatty acid synthase provides a survival advantage to colorectal cancer cells via upregulation of cellular respiration

**DOI:** 10.18632/oncotarget.3783

**Published:** 2015-04-20

**Authors:** Yekaterina Y. Zaytseva, Jennifer W. Harris, Mihail I. Mitov, Ji Tae Kim, D. Allan Butterfield, Eun Y. Lee, Heidi L. Weiss, Tianyan Gao, B. Mark Evers

**Affiliations:** ^1^ Markey Cancer Center, University of Kentucky, Lexington, Kentucky, USA; ^2^ Department of Surgery, University of Kentucky, Lexington, Kentucky, USA; ^3^ Department of Chemistry, University of Kentucky, Lexington, Kentucky, USA; ^4^ Pathology and Laboratory Medicine, University of Kentucky, Lexington, Kentucky, USA

**Keywords:** FASN, colorectal cancer, energy homeostasis, metastasis, metabolic stress

## Abstract

Fatty acid synthase (FASN), a lipogenic enzyme, is upregulated in colorectal cancer (CRC). Increased *de novo* lipid synthesis is thought to be a metabolic adaptation of cancer cells that promotes survival and metastasis; however, the mechanisms for this phenomenon are not fully understood. We show that FASN plays a role in regulation of energy homeostasis by enhancing cellular respiration in CRC. We demonstrate that endogenously synthesized lipids fuel fatty acid oxidation, particularly during metabolic stress, and maintain energy homeostasis. Increased FASN expression is associated with a decrease in activation of energy-sensing pathways and accumulation of lipid droplets in CRC cells and orthotopic CRCs. Immunohistochemical evaluation demonstrated increased expression of FASN and p62, a marker of autophagy inhibition, in primary CRCs and liver metastases compared to matched normal colonic mucosa. Our findings indicate that overexpression of FASN plays a crucial role in maintaining energy homeostasis in CRC via increased oxidation of endogenously synthesized lipids. Importantly, activation of fatty acid oxidation and consequent downregulation of stress-response signaling pathways may be key adaptation mechanisms that mediate the effects of FASN on cancer cell survival and metastasis, providing a strong rationale for targeting this pathway in advanced CRC.

## INTRODUCTION

The metabolic properties of cancer cells are remarkably different from those of normal cells [1]. An increased rate of lipid synthesis in cancer has been recognized as an important aspect of metabolism in transformed cells [2]. In contrast to normal epithelial cells, the majority of fatty acids in malignant cells are derived from *de novo* lipogenesis, regardless of the availability of extracellular lipids [3, 4]. Fatty acid synthase (FASN), a key enzyme of *de novo* lipid biosynthesis [5], is significantly unregulated in many cancers, including colorectal cancer (CRC) [6–8], and is associated with aggressive disease and a poor prognosis [9, 10]. Previously, we showed that the expression of FASN progressively increases with increased CRC stage [10]. Furthermore, our *in vivo* studies demonstrated that shRNA-mediated inhibition of FASN significantly reduces lung and hepatic metastases in nude mice and inhibits angiogenesis in an orthotopic CRC mouse model [9, 10]. Consistent with our findings, other studies have shown an association of *de novo* lipid synthesis with metastatic prostate cancer and melanoma [11, 12].

Reprogrammed energy metabolism is a hallmark of cancer cells and is rapidly emerging as a potential target for therapeutic intervention [13–15]. The ability to overcome metabolic stress is a crucial step for cancer cell survival and metastasis [16]. Upregulation of lipid synthesis has been identified as a metabolic adaptation that promotes cancer cell survival; however, the exact mechanisms involved in this adaptation are not completely understood [3, 17]. Furthermore, even though there is clear evidence that the energy status of tumor cells is crucial for maintenance of the transformed phenotype and metastatic capabilities [18, 19], the role of FASN in the regulation of energy homeostasis in cancer cells is not yet established. Fatty acids are energy-providing substrates catabolized by fatty acid oxidation (FAO) [18]. Recent studies suggest that when cancer cells require additional adenosine triphosphate (ATP), FAO is critically important for cell survival [20–22]. However, it remains unclear whether cancer cells preferentially oxidize exogenously-derived fatty acids or favor the oxidation of endogenous fatty acids, which are synthesized at a high rate by FASN.

To overcome metabolic stress, cancer cells activate several pro-survival pathways. Activation of AMP-activated protein kinase (AMPK), an established metabolic stress sensor, occurs with even modest decreases in ATP production. This activation promotes enhanced activity of catabolic pathways that generate more ATP and inhibits anabolic pathways [23]. Autophagy also represents a crucial mechanism which allows tumor cells to adjust to changes in nutrient availability [24]. However, the link between autophagy and *de novo* lipid synthesis has not been established. In the present study, we test the hypothesis that overexpression of FASN promotes a switch in metabolic pathways that drives cellular bioenergetics along routes that support cancer cell survival during CRC progression. We show that overexpression of FASN leads to a significant increase in cellular respiration including enhanced FAO. Consistently, we show that under conditions of energy stress, high expression of FASN is associated with a lower level of AMPK activation and p62 accumulation, a marker of autophagy inhibition. Collectively, our data suggest that upregulation of *de novo* lipogenesis is protective to CRC cells during energy stress conditions and, thus, can play a crucial role in cancer progression and metastasis.

## RESULTS

### FASN regulates cellular respiration in CRC

**Figure 1:**
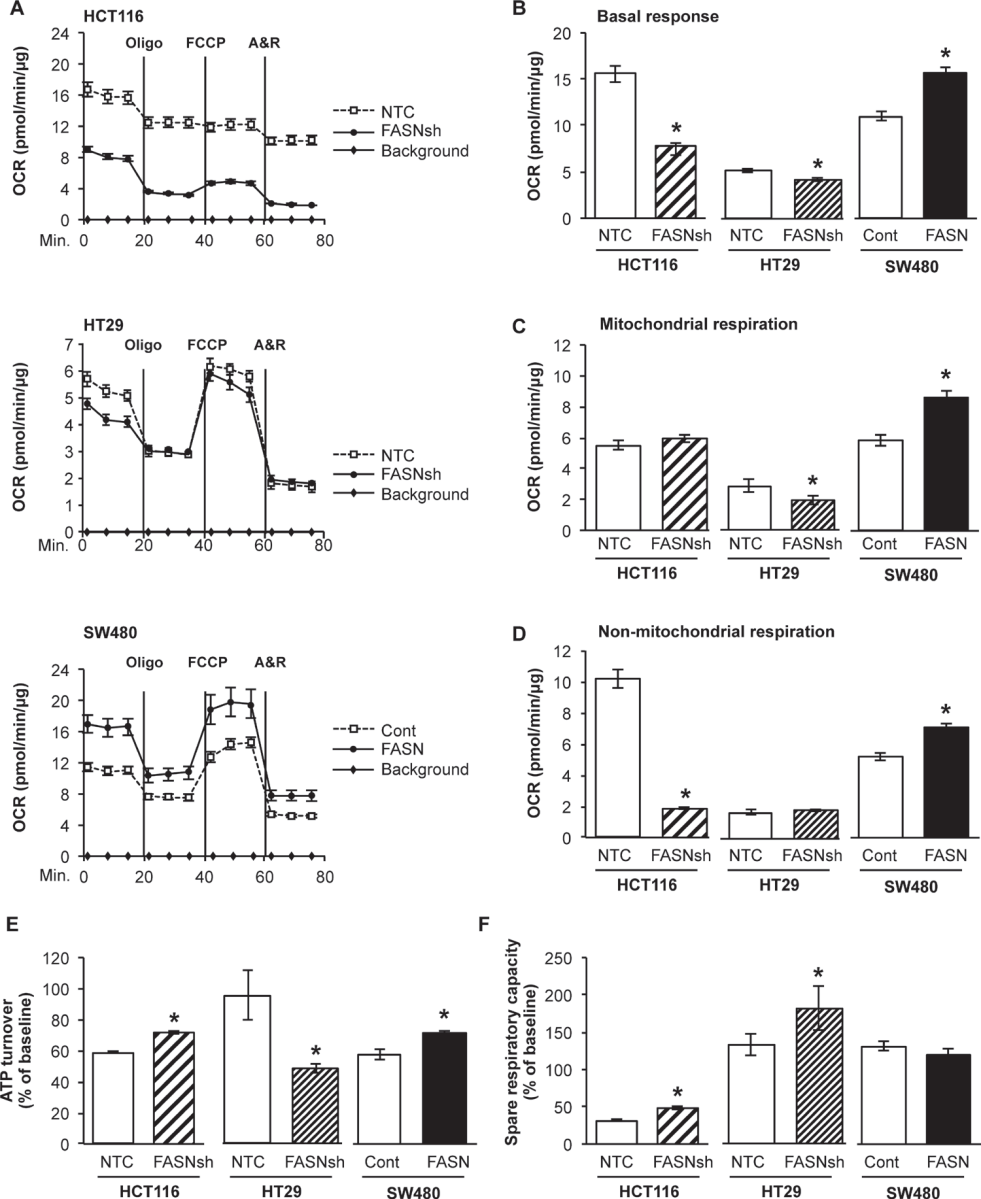
Fatty acid synthase regulates cellular respiration in CRC cell lines **A.** Cellular respiration of HCT116, HT29 and SW480 cells with altered expression of FASN was analyzed by mitochondrial stress test using a Seahorse XF96 Analyzer. Oxygen consumption rate (OCR) was measured prior to and after injections of oligomycin (Oligo) at 1 μM working concentration, carbonyl cyanide-4-(trifluoromethoxy) phenylhydrazone (FCCP) at 0.6 μM working concentration and combination of antimycin A and rotenone (A&R) at 1 μM working concentration (see Materials and Methods). Representative graphs are shown. **B-F.** Data analysis was performed using Wave 2.1 (Seahorse Bioscience). Quantitative analysis demonstrates differences in B. basal response (Measurements 1–3), C. mitochondrial respiration, and D. non-mitochondrial respiration (OCR after antimycin A and rotenone exposure) upon changes in expression of FASN in HCT116, HT29 and SW480 cell lines. E. ATP turnover is expressed as a percentage of a baseline value and calculated as 100 × (OCR phase 1 − OCR phase 2) / Basal OCR in CRC cell lines. F. Spare respiratory capacity is expressed as a percentage of a baseline value and calculated as 100 × (OCR phase 3 − OCR phase 4) / Basal OCR. All experiments were performed at least twice using multiple replicates; *p < 0.05.

To sustain uncontrolled proliferation and survive energy stress conditions during cancer progression, cancer cells alter their energy production [25]. To evaluate the effect of FASN on cellular respiration, oxygen consumption rate (OCR) was measured in HCT116 and HT29 CRC cell lines with stable knockdown of FASN and in SW480 cells with stable overexpression of FASN using the Seahorse XF Extracellular Flux Analyzer (FASN expression in CRC cell lines is shown in Supplemental Figure 1). High levels of FASN are associated with a significant increase in basal response (including both mitochondrial and non-mitochondrial respiration) of CRC cell lines under standard mitochondrial stress assay conditions (Figure 1A-B). Quantification of mitochondrial and non-mitochondrial respiration showed that the lower cellular respiration in HCT116 cells with stable knockdown of FASN, as compared to control, is primarily due to a decrease in non-mitochondrial respiration of cells; however, knockdown of FASN in HT29 cells predominantly decreased mitochondrial respiration. Overexpression of FASN significantly increased both mitochondrial and non-mitochondrial respiration in SW480 cells (Figure 1C-D). Consistently, knockdown of FASN in HT29 cells was associated with decreased energy production by mitochondria, as shown by ATP turnover for mitochondrial respiration, whereas overexpression of FASN in SW480 cells increased ATP turnover. Interestingly, stable knockdown of FASN in HCT116 cells, which, as we found, heavily rely on glycolysis, significantly increased mitochondrial turnover of ATP (Figure 1E). These findings suggest a possible compensatory mechanism by cancer cells with reduced ATP levels to upregulate mitochondrial activity. We also observed an increase in spare respiratory capacity of mitochondria HCT116 and HT29 cells with knockdown of FASN, but no significant change in respiratory capacity was noted when FASN was overexpressed in SW480 cells (Figure 1F). Together, these data suggest that FASN plays an important role in regulation of energy homeostasis in CRC.

### Inhibition of FASN decreases the dependence of CRC cells on glycolysis

**Figure 2:**
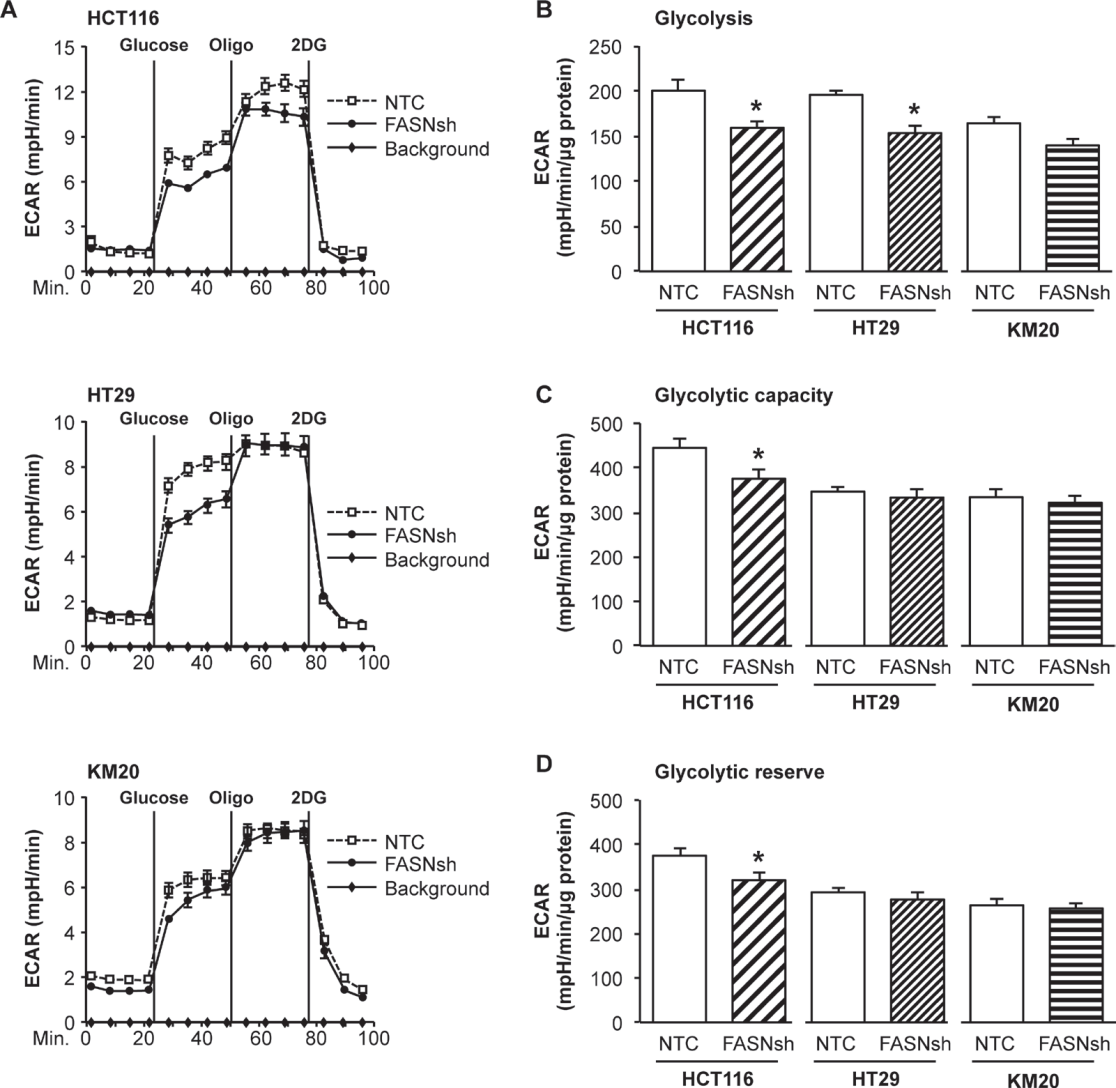
Inhibition of FASN decreases glycolysis in CRC cells **A.** Representative graphs of XF Glycolysis Stress Test using CRC cell lines with stable knockdown of FASN. Extracellular acidification rate (ECAR) is used as a measure of anaerobic glycolysis. **B.** Quantitative analysis of glycolysis, **C.** glycolytic capacity, and **D.** glycolytic reserve in NTC and FASNsh CRC cell lines. Data shown as an average of area under the curve and calculated using ANOVA and Tukey post test (XF Glycolysis Test Software). All experiments were performed at least twice using multiple replicates; *p < 0.05.

Many cancer cells rely on aerobic glycolysis for growth and survival [15]. To confirm our data from the mitochondrial stress test indicating that the glycolytic activity of CRC cells may depend on the level of FASN expression, we performed a glycolysis stress test. As demonstrated in Figure 2A-B, stable inhibition of FASN in HCT116 and HT29 CRC cell lines led to a significant decrease in glycolysis. Similar results were observed in KM20 cells; however, the results were not statistically significant (Figure 2A-B). Consistent with the high reliance of HCT116 cells on glycolysis, we found that inhibition of FASN significantly decreased the glycolytic capacity and glycolytic reserve in this cell line. In contrast, no difference in these parameters was observed in the other tested cell lines (Figure 2C-D). Together, these results suggest that upregulation of FASN contributes to maintaining elevated glycolysis in CRC cells.

### FASN regulates the level of FAO under different conditions of substrate availability

**Figure 3:**
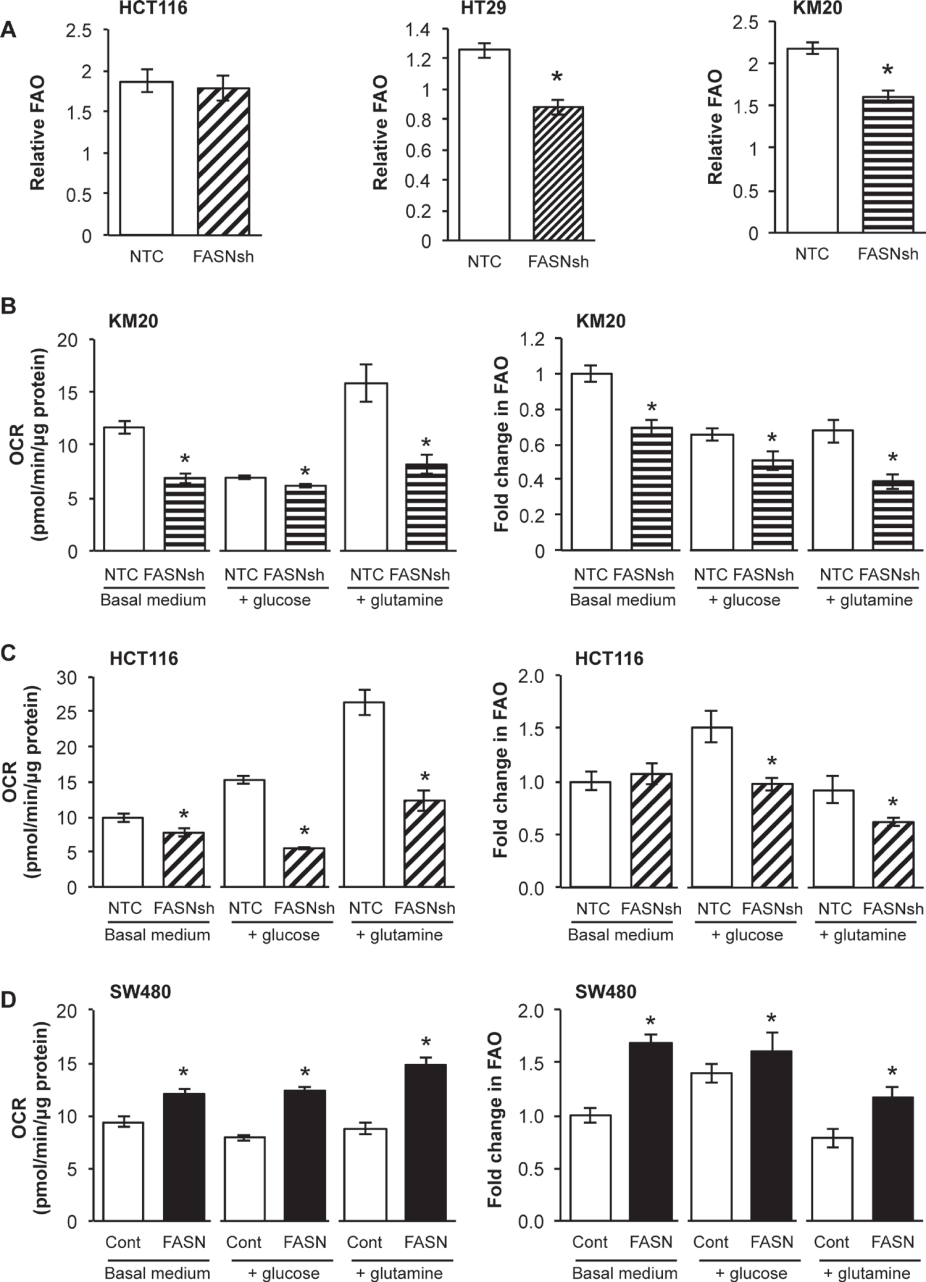
FASN regulates the level of FAO under different conditions of substrate availability OCR was measured in cells in the absence or presence of ETO (40 μM) using a Seahorse XF96 Analyzer. FAO was quantified as a response to ETO treatment. **A.** Changes in FAO in CRC cells with stable knockdown of FASN. Experiments performed in FAO assay medium (2.5 mm glucose, 0.5 mm carnitine, 5 mm HEPES). **B-D.** OCR and FAO of NTC and FASN knockdown KM20 (B), NTC and FASN knockdown HCT116 (C), and control and FASN overexpression SW480 (D) cells were measured while cells cultured on no substrate medium, medium supplemented with glucose (25 mM) or with glutamine (6 mM). Data analysis was performed using Wave 2.1 (Seahorse Bioscience). All experiments were performed at least twice using multiple replicates; *p < 0.05.

Mitochondrial respiration burns glucose, fatty acids, and amino acids to produce usable energy [26]. Fatty acids are degraded in mitochondria by β-oxidation and oxidative phosphorylation to produce ATP [18]. However, it remains unknown whether cancer cells preferentially oxidize endogenous fatty acids or utilize exogenous fatty acids as substrates for energy production. To test whether *de novo* synthesized fatty acids contribute to FAO, OCR was measured in cells in the absence or presence of etomoxir (ETO), an inhibitor of carnitine palmitoyltransferase-1 (CPT-1). CPT-1 is a rate-limiting step of the CPT system, which is required for transport of fatty acids to the mitochondrial matrix for oxidation [27]. Therefore, inhibition of CPT-1 should decrease OCR of cells if endogenous fatty acids are oxidized [26]. Figure 3A demonstrates that stable knockdown of FASN significantly decreased FAO in HT29 and KM20 cells, but not in HCT116 cells.

To assess oxidation of exogenous fatty acids in control and FASN knockdown CRC cells, we utilized the XF Palmitate-BSA FAO substrate assay. Interestingly, supplementation of exogenous palmitate uncoupled the electron transport and phosphorylation reactions, and thus inhibited production of ATP in both control and FASN knockdown HCT116 cells. Furthermore, in this experimental setting, we observed that inhibition of FASN in HCT116 cells decreased oxidation of both endogenous fatty acids and exogenously derived palmitate as compared with control cells (Supplemental Figure 2). Similar results were observed in HT29 cells (data not shown).

To extend investigations on the role of FASN in mitochondrial respiration, we measured OCR and quantified FAO in cells cultured with no substrate medium, or medium supplemented with glucose or glutamine. We observed a significant decrease in OCR and FAO in KM20 cells with stable knockdown of FASN cultured in basal medium or medium supplemented with either glucose or glutamine as compared with control cells (Figure 3B). Inhibition of FASN in HCT116 cells cultured under similar conditions also led to a significant decrease in OCR, particularly when the medium was supplemented with glucose or glutamine (Figure 3C). We did not see a significant difference in FAO in HCT116 cells with stable knockdown of FASN as compared to control cells cultured in basal medium. However, when cultured in medium supplemented with glucose or glutamine, HCT116 cells with stable knockdown of FASN utilized FAO to a significantly less degree as compared with control cells (Figure 3C). In contrast, overexpression of FASN in SW480 cells significantly increased OCR and FAO when cells were cultured in basal medium or in medium supplemented with either glucose or glutamine (Figure 3D). Collectively, these results suggest that CRC cells, under a variety of substrate availabilities, efficiently oxidize *de novo* synthesized fatty acids.

### Overexpression of FASN significantly increases dependence of CRC cells on FAO in matrix-detached conditions

**Figure 4:**
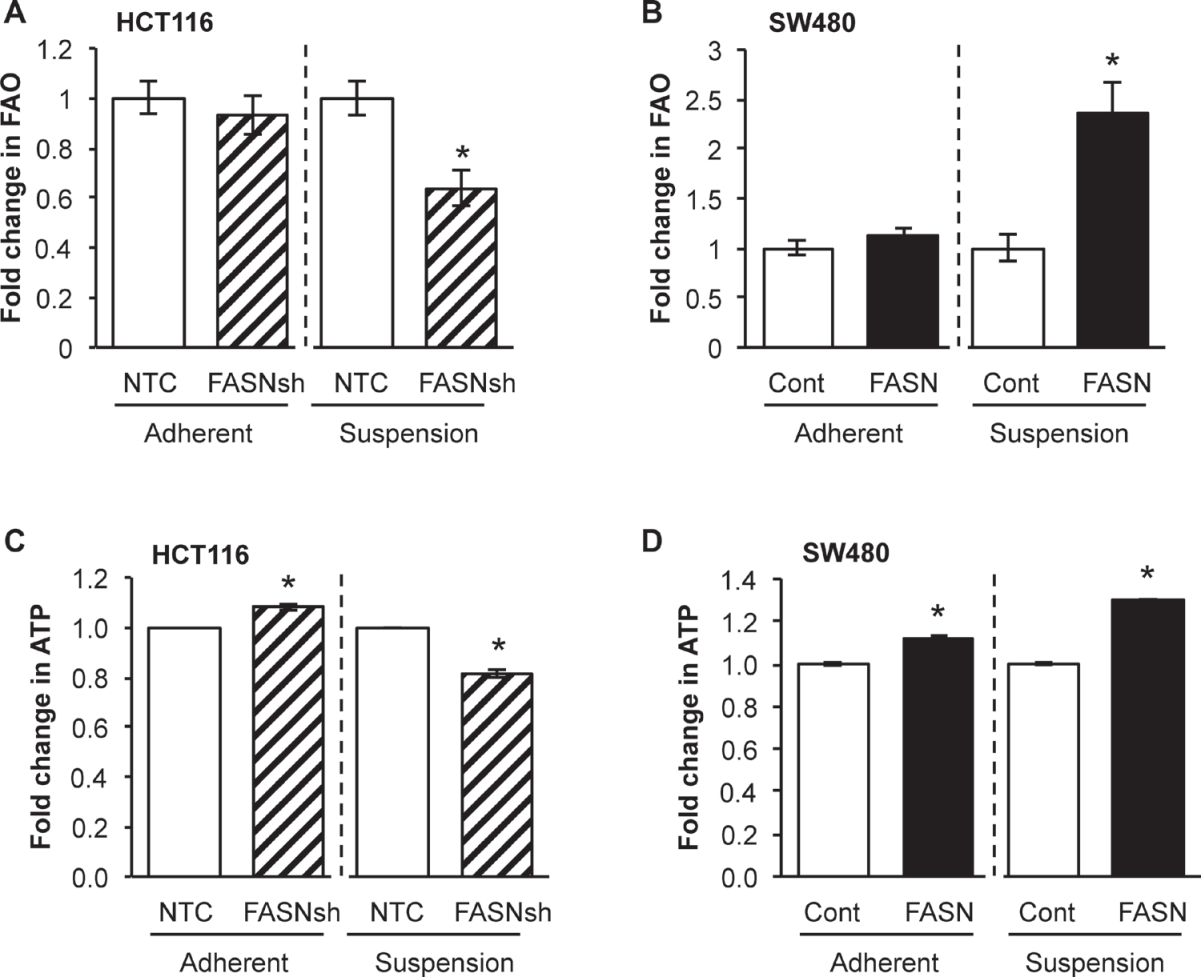
FASN overexpression significantly increases dependence of CRC cells on FAO in matrix-detached conditions **A.** Fold change in FAO in NTC and FASN knockdown HCT116 cells in adherent and matrix-detached conditions. **B.** Fold change in FAO in control and FASN overexpression SW480 cells in adherent and matrix-detached conditions. **C.** Fold change in the production of ATP in NTC and FASN knockdown HCT116 cells in adherent and matrix-detached conditions. **D.** Fold change in the production of ATP in control and FASN overexpression SW480 cells in adherent and matrix-detached conditions. All experiments were performed at least twice using multiple replicates; *p < 0.05.

Similar to glucose starvation, matrix detachment elicits energy stress [16, 18]. Upregulation of UCP2, a key regulator of mitochondrial FAO [28], observed in CRC cells when cultured in matrix-detached conditions, supports our hypothesis that CRC cells extensively utilize FAO under these conditions (data not shown). To elucidate the role of FASN in FAO and production of ATP in a matrix-detached condition, OCR was measured in cells seeded onto XF cell culture plates pre-coated with poly 2-hydroxyethyl methacrylate (Poly-HEMA). Inhibition of FASN in HCT116 cells significantly decreased FAO with matrix detachment (Figure 4A); in contrast, overexpression of FASN in SW480 cell lines significantly increased FAO (Figure 4B). Consistently, the high level of FASN in these cell lines was associated with an increased production of ATP in these conditions (Figure 4C-D). Together, these results suggest that overexpression of FASN is associated with increased FAO in matrix-detached conditions and is consistent with increased production of ATP by CRC cells.

### Upregulation of FASN in CRC cells is associated with a decrease in activity of AMPK and increased expression of p62

**Figure 5:**
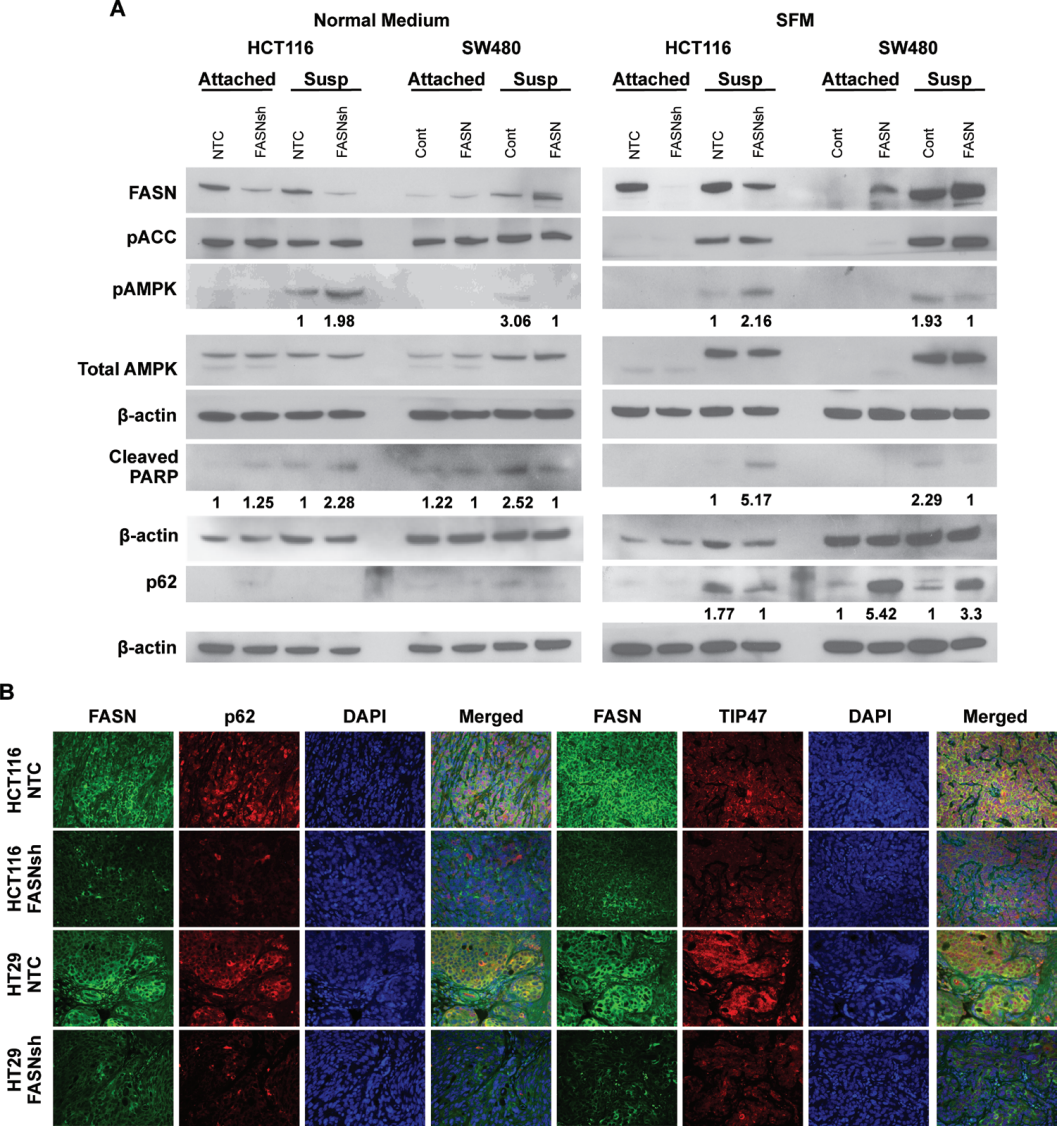
Upregulation of FASN in CRC cells is associated with a decrease in activity of AMPK and accumulation of p62 **A.** Western blot analysis of CRC cell lines with altered expression of FASN for pAMPK, total AMPK, p62, and cleaved PARP cultured in adherent and matrix-detached conditions on either normal or serum free medium for 48 h. The relative expression of pAMPK, cleaved PARP and p62 was quantified by normalizing band intensity to total AMPK and actin and is shown under the corresponding bands as a fold change between NTC vs FASNsh or control vs FASN. **B.** Immunofluorescent staining of tissue sections from orthotopic CRCs (NTC and FASNsh HCT116; NTC and FASNsh HT29) for FASN (green), p62 (red), TIP47 (red), and 4′,6-diamidino-2-phenylindole (DAPI) (blue). x40 magnification.

AMPK is a crucial sensor of energy status and is activated in response to declining fuel supply [29]. From a metabolic standpoint, AMPK regulates energy homeostasis under conditions of metabolic stress by activating pathways of catabolic metabolism such as autophagy [29, 30]. To test whether changes in the level of FASN expression and bioenergetic status of CRC cells correspond to changes in major energy-sensing pathways, HCT116 cells with stable knockdown of FASN and SW480 cells with stable overexpression of FASN were cultured in matrix-attached or matrix-detached conditions for 48 h prior to western blot analysis. Stable knockdown of FASN induces apoptosis in CRC cells cultured either in normal or serum free medium (SFM) for 48 h (Supplemental Figure 3). As shown in Figure 5A, stable inhibition of FASN expression in HCT116 cells is associated with an increased phosphorylation of AMPK when cells are cultured in either normal medium or SFM in matrix-detached conditions; in contrast, overexpression of FASN in SW480 cells decreased activity of pAMPK in these conditions. Furthermore, knockdown of FASN expression in HCT116 is associated with downregulation of p62 expression, a marker of autophagy inhibition, when cultured in SFM in matrix-detached conditions. In contrast, overexpression of FASN in SW480 cells led to a marked increase in expression of p62 in SFM in both attached and matrix-detached conditions. We also noted an increase in PARP cleavage in HCT116 cells with FASN knockdown as compared to control when cells were cultured in normal medium in attached and matrix-detached conditions or in SEM in matrix-detached conditions. Culturing SW480 cells with overexpression of FASN under the same conditions led to a decrease in PARP cleavage (Figure 5A).

To test whether the levels of other autophagy markers are regulated by FASN, we assessed expression of microtubule-associated protein 1 light chain 3 (LC3) and Beclin 1, the autophagosome-associated proteins [31, 32], by western blot analysis. As shown in Supplemental Figure 4A, culturing HCT116 and SW480 cells in serum free, matrix-detached conditions significantly increased expression of Beclin1, suggesting that the combination of nutrient starvation and matrix-detachment induces autophagy; however, no significant difference in Beclin 1 was observed due to altered expression of FASN in any experimental conditions. Surprisingly, analysis of LC3B expression, one of the isoforms of LC3 commonly used as an autophagy marker [31], was not consistent with induction of autophagy when cells were cultured in serum free, matrix-detached conditions; however, an increase in expression of this marker was observed when SW480 cells overexpressing FASN were cultured in normal medium. Interestingly, expression of LC3A, another isoform of LC3, was consistently induced by cell starvation or matrix-detachment in HCT116, HT29 and SW480 cell lines. Moreover, inhibition of FASN in HCT116 and HT29 cells significantly induced expression of LC3A, whereas overexpression of FASN in SW480 cells inhibited expression of this protein (Supplemental Figure 4B-C). To confirm these results, we assessed the level of LC3A in HCT116 and HT29 cells with stable knockdown of FASN using confocal microscopy. As shown in Supplemental Figure 4D, inhibition of FASN in HCT116 and HT29 cells, cultured in SFM, increases expression of LC3A, suggesting that a decrease in expression of FASN induces autophagy in CRC cells.

To test whether FASN regulates expression of p62 *in vivo*, we analyzed expression of p62 in orthotopic CRCs established by implantation of HCT116 and HT29 cells, control and FASN knockdown, into colonic submucosa of athymic mice using murine colonoscopy as previously described [9]. As demonstrated by double immunofluorescent staining for FASN and p62, stable inhibition of FASN in HCT116 and HT29 orthotopic CRCs is associated with a decrease in expression of p62 (Figure 5B).

Lipid droplet accumulation is a frequent phenomenon of cancers, including CRC [33]. We assessed the expression of tail-interacting protein of 47kDa (TIP47), a lipid droplet-associated protein, in the orthotopic CRCs. As shown in Figure 5B, inhibition of FASN in HCT116 and HT29 orthotopic tumors is associated with a decrease in expression of TIP47, suggesting that inhibition of FASN diminishes accumulation of lipid droplets in CRC. Interestingly, further analysis of FASN, p62 and TIP47 staining in orthotopic CRCs using confocal microscopy revealed a high degree of spectral overlap between FASN and p62, and FASN and TIP47, suggesting that these proteins may reside in close proximity or interact in CRC cells (Supplemental Figure 4D).

Collectively, these results suggest that, under stress conditions, overexpression of FASN protects cancer cells by dampening signaling triggered by energy stress.

### Expression of FASN significantly correlates with the level of p62 expression in primary CRC

**Figure 6:**
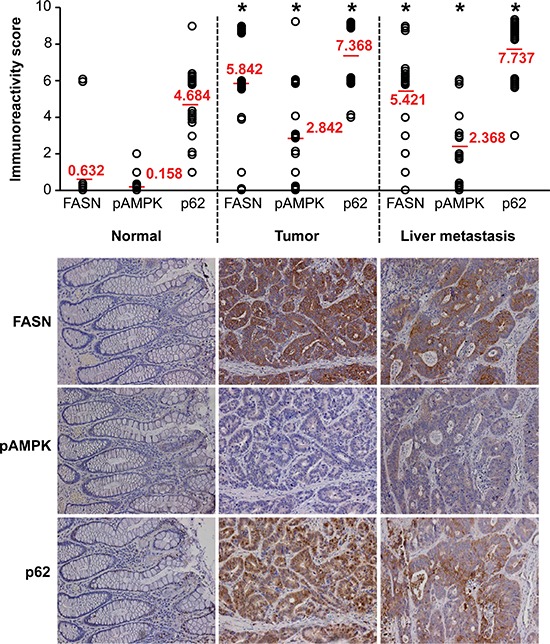
Expression of FASN significantly correlates with expression of p62 in primary CRC Expression of FASN, pAMPK, and p62 was analyzed in human tissues (matching primary CRC, liver metastasis, and normal colon), *n* = 19, using IHC. Immunoreactivity score was determined by multiplication of the values for staining intensity (0, no staining; 1, weak staining; 2, moderate staining; 3, strong staining) and the values for percentage of positive tumor cells (0, no positive cells; 1, 0–10% positive; 2, 11–50% positive; 3, 51–100% positive); *p < 0.001 as compared to normal tissue.

We have previously demonstrated that, compared to normal colonic mucosa, the level of FASN expression is significantly higher in primary CRC and liver metastases [10]. Using immunohistochemical (IHC) analysis, expression of FASN, pAMPK and p62 was assessed in clinical samples, which included matched primary cancer, liver metastases and normal colonic mucosa from 19 CRC patients. Statistical evaluation of immunoreactivity scores showed significantly increased expression of FASN, pAMPK and p62 in primary CRC and liver metastases as compared with normal colon mucosa. However, no significant difference in expression for these markers was noted between the primary cancer and matching liver metastasis (Figure 6). We also observed a moderate correlation between FASN and p62 that was statistically significant (Spearman's *r* = 0.46, *p* = 0.049); no other significant correlations were noted. Western blot and IHC analyses of 5 additional CRC surgical specimens, which included primary CRC and matched normal colonic mucosa, were consistent with IHC analysis of clinical samples and showed that high expression of FASN is associated with an increased expression of p62 in primary CRC (Supplemental Figure 5).

## DISCUSSION

Overexpression of FASN has been shown to promote cancer growth and metastasis [9, 10, 17, 34]. Lipids endogenously synthesized by FASN can be utilized for the synthesis of phospholipids and as constituents for membrane biosynthesis; however, its role in providing substrates for energy production has not yet been established [26]. Here, for the first time, we provide a comprehensive analysis of CRC cell metabolism regarding its dependence on expression of FASN. First, we show that a high level of FASN expression can contribute to enhanced glycolysis and mitochondrial respiration in CRC. Second, we demonstrate that a high level of FASN expression is associated with increased FAO in CRC cells, particularly during metabolic-stress conditions. Consistently, we demonstrate that overexpression of FASN prevents activation of stress-sensing pathways, such as the AMPK signaling pathway and autophagy, and leads to accumulation of lipid droplets in CRC.

Recent studies have shown that, despite enhanced glycolysis, mitochondrial oxidative phosphorylation is functionally intact in most cancers, and each cancer has unique metabolic features [35]. Consistently, quantitative analysis of metabolism of CRC cell lines, presented in our study, revealed that their reliance on glycolysis and mitochondrial respiration is cell line dependent and most likely dependent on the genetic background and mutational status of the cancer cells. As an example, the analysis of cellular respiration in HCT116 and HT29 demonstrates that HCT116 cells predominantly rely on non-mitochondrial respiration including glycolysis as compared to HT29 cells, which are highly dependent on the mitochondrial pathway for production of ATP. Furthermore, we noted that a decrease in glycolysis due to inhibition of FASN in HCT116 increases mitochondrial respiration, thus providing evidence of the interplay between these energy pathways. The utilization of FAO by HCT116 cells in the presence of glucose and glutamine further supports the metabolic flexibility of cancer cells to adapt the mechanisms of energy production to changes in the substrate availability. Interestingly, despite the genetic and metabolic diversity of cell lines used in this study and, inhibition of FASN led to a significant decrease in cellular respiration in all cell lines, whether due to inhibited glycolysis, mitochondrial respiration, or both, suggesting that inhibition of *de novo* lipogenesis would have a beneficial effect in a wide range of tumors. The effect of FASN in sustaining an elevated glycolytic activity of cancer cells can be explained by several mechanisms. First, it is possible that lipid synthesis-derived NADP^+^ could help to increase the availability of cytosolic NAD^+^ required to maintain glycolysis [33, 36]. Furthermore, limited mitochondrial ATP production can impair glycolytic ATP synthesis through a decrease in the mitochondria-bound hexokinase activity [26]. Therefore, the decrease in glycolysis observed in HT29 and KM20 cells with stable inhibition of FASN could be a secondary effect mediated by a decrease in ATP production in mitochondria. Additional investigation is required to fully understand the FASN-mediated mechanisms that regulate glycolytic activity in CRC cells.

Fatty acids represent an important energy source which produce twice as much ATP as carbohydrates [18]. The previous dogma that concurrent activities of fatty acid synthesis (FAS) and FAO are incompatible has been revised by discoveries that FAS and FAO can function both simultaneously and independently from each other [16, 18, 37]. In the present study, we demonstrate that upregulation of FASN enhances the activity of FAO and increases production of ATP, particularly during metabolic stress. In agreement with these findings, recent studies suggest that FAO may be crucial to support cancer cell survival during periods of energy stress [21, 22]. Furthermore, activation of FAO is implicated in maintaining cancer cell viability, production of ATP, and resistance to oxidative stress in glioblastoma cells [26] and is a key mechanism of pancreatic cell survival during tumor relapse [38].

Glucose-derived carbon is considered the primary source for *de novo* lipid synthesis [26]. Similarly, cancer cells are extremely sensitive to glutamine deprivation; therefore, glutamine could be a significant contributor to FAS [39]. Consistently, our results show that inhibition of FASN in CRC cells cultured on different substrates leads to a decrease in cellular respiration including FAO, thus confirming that both glucose and glutamine contribute to *de novo* lipogenesis in CRC even though the efficiency of utilization of these substrates is cell line dependent. We also investigated whether the level of FASN expression regulates oxidation of exogenously derived fatty acids. Using fluorescently labeled palmitate, we found no change in uptake of exogenous fatty acids when expression of FASN was inhibited (data not shown); however, FASN knockdown in HCT116 and HT29 cells prevented oxidation of both endogenous and exogenous fatty acids. We did not investigate the mechanism behind this phenomenon in the present study, but our recent data suggest that stable inhibition of FASN in CRC cells may change the expression of proteins, such as CPT-1, which are involved in transport of fatty acids to mitochondria and regulated by intermediate substrates of *de novo* lipogenesis [40].

Cancer cells have multiple systems to sense energy balance and can modify signaling pathways to ensure survival under conditions of metabolic stress [13]. The pharmacological inhibition of FASN with C93, a synthetic FASN inhibitor, causes rapid energy depletion and activation of AMPK, which leads to cytotoxicity, in ovarian cancer cells [41]. Consistently, results of our present study support the hypothesis that overexpression of FASN is protective during metabolic stress by satisfying the energy demand necessary for CRC cells to survive and support their functions. Activation of AMPK in FASN knockdown CRC cells indicates that these cells are under more stress than control cells. In contrast, CRC cells with overexpression of FASN appear to be exposed to less stress as indicated by the lower level of phosphorylated AMPK. Interestingly, AMPK is also known as a negative regulator of glycolysis [29]. Therefore, low AMPK activity in CRC cells with high FASN expression might also explain the increased glycolysis in these cells. A recent study shows that pharmacological activation of AMPK suppresses *de novo* lipogenesis in prostate cancer [42], suggesting the role of AMPK as an upstream regulator of lipid synthesis. Since both FASN and AMPK have been actively evaluated as targets for cancer therapy in pre-clinical and clinical trials, a better understanding of the interconnection between these proteins is necessary in order to develop more advanced therapeutic strategies.

p62 was identified as one of the specific substrates that is degraded during autophagy [43, 44]. In this study, we identified a novel link between *de novo* lipogenesis and p62, showing that overexpression of FASN is associated with p62 accumulation not only in CRC cell lines exposed to metabolic stress, but also in orthotopic human CRCs *in vivo* and in clinical samples. We identified a moderate but significant correlation between FASN and p62 expressions in primary CRC. In agreement with our study, increased accumulation of p62 was associated with aggressive clinicopathologic features and an unfavorable prognosis in oral squamous cell carcinoma [45] and in lung adenocarcinoma [46]. Analysis of the correlation between FASN and p62 expression in liver metastases did not reach statistical significance suggesting that an increased sample size should be considered in future evaluations.

The connection between autophagy and p62 is complex and, in some cases, conflicting. Despite our observation that an increase in accumulation of p62 is associated with a decrease of expression of the autophagosomal marker, LC3A, and with a significant increase in lipid droplets, thus supporting the model that overexpression of FASN is associated with autophagy inhibition, a comprehensive investigation is required to better understand the role of FASN in autophagy. Furthermore, recent studies demonstrate that, in addition to autophagy, p62 plays a crucial role in a number of cellular processes related to malignant transformation and tumor progression [43, 47]; therefore, FASN-induced accumulation of p62 may also be independent of autophagy in the progression of CRC. We are currently pursuing studies to further define the role of FASN-mediated upregulation of p62 in CRC.

In summary, this study is the first to provide insight into metabolic changes that occur in cancer cells upon upregulation of *de novo* lipogenesis. Importantly, we have shown that endogenously synthesized FASN lipids are utilized for β-oxidation in CRC cells, particularly under energy stress conditions, suggesting that activation of FASN can be a crucial mechanism that sustains cancer cell survival and prevents cell death during progression of CRC and metastasis. FAO has been implicated in drug resistance, protection from oxidative stress, inhibition of pro-apoptotic pathways, supplementation of metabolic intermediates for cell growth, and in tumor relapse [18, 38]. Therefore, inhibition of *de novo* lipogenesis may represent a potential strategy for overcoming resistance to chemotherapeutic agents and radiation, and also as a possible preventative treatment for metastatic CRC.

## MATERIALS AND METHODS

### Cell lines

CRC lines HCT116, HT29, KM20 and SW480 were authenticated November 2011 (Genetica DNA Laboratories, Cincinnati, OH). Stable FASN knockdown CRC cell lines were established and lipid biosynthesis was assessed as previously described [10]. FASN cDNA (ID6172538, Open Biosystem, Chicago, IL) was cloned into the pEGFP vector. Stable overexpression was established by transfecting SW480 cells with PEGFP-FASN vector with Gentamicin (Invitrogen, Austin, TX) selection as previously described [9].

### Measurement of cellular oxygen consumption rate, extracellular acidification rate, and FAO using the XF96 extracellular flux analyzer

Cellular oxygen consumption rate and extracellular acidification rate (ECAR) were used to monitor basal respiration, mitochondrial respiration, glycolysis, and FAO in real time. Cell concentrations for XF96 plates (Seahorse Bioscience, North Billerica, MA) varied by cell type: HCT116 was 1.5 × 10^4^ cells/well; SW480 was 2.0 × 10^4^ cells/well; HT29 was 3.0 × 10^4^ cells/well; and, KM20 was 4.0 × 10^4^ cells/well. Plated cells were maintained for 24 h in a 37°C/5% CO_2_ incubator. One hour prior to assay, cells were equilibrated with bicarbonate-free low buffered DMEM medium without any supplement, or supplemented with glucose or glutamine as indicated, in a 37°C non-CO_2_ incubator. Reagents required for the experimental protocol were prepared in assay medium, loaded into reservoirs and automatically injected into plates as required for each assay.

### XF Cell Mito Stress Test

Experimental design followed the protocol provided by Seahorse Bioscience. Briefly, cells were metabolically perturbed by the addition of three different compounds in succession and the OCR was measured prior to and after injection of each compound. First, cells were injected with oligomycin (1 μM working concentration), which inhibits ATP synthesis and identifies the percentage of OCR devoted to ATP synthesis. The second compound, carbonyl cyanide-4-(trifluoromethoxy) phenylhydrazone (FCCP) (0.6 μM working concentration), an uncoupling agent, collapses the mitochondrial membrane potential; a rapid consumption of energy and oxygen without ATP generation follows. FCCP was used to calculate the maximum and spare respiratory capacity of cells. Finally, cells were exposed to a combination of rotenone, a complex I inhibitor, and antimycin A, a complex III inhibitor (1 μM working concentration). This combination inhibits mitochondrial respiration and allows calculation of the mitochondrial and non-mitochondrial fractions contributing to respiration.

### XF Glycolysis Stress Test

For the first ECAR assessment, non-glycolytic acidification was obtained in the absence of extracellular glucose and pyruvate. Next, cells were exposed to three compounds, with measurements after each injection. The first injection, a saturating concentration of glucose (10 mM), causes an increase in ECAR and defines the rate of glycolysis under basal conditions. The second injection, oligomycin (1 μM working concentration), inhibits mitochondrial respiration and shifts energy production toward glycolysis, revealing the maximum glycolytic capacity of the cells. The final injection, 2-deoxy-D-glucose (2-DG; 100 mM), a glucose analog, inhibits glycolysis. The difference between glycolytic capacity (Measurement 2) and glycolysis (Measurement 1) defines the glycolytic reserve.

### Fatty acid oxidation (FAO)

Fatty acid oxidation was determined by monitoring the OCR of cells with no exogenous glucose or glutamine and by using a specific FAO inhibitor, etomoxir (40 μM). FAO was quantified as a response to ETO treatment as previously described [26]. The XF Palmitate - BSA FAO substrate (Seahorse Bioscience) was used to determine the ability of cells to oxidize exogenously added fatty acids, and the proportion of respiration that is supprted by exogenous fatty acids. The substrates were prepared and experiments performed according the manufacturer's protocol (part #102720-100 Seahorse Bioscience). For measurement of OCR and FAO in suspension culture, XF96 cell culture plates were coated with Poly-HEMA (Sigma Cat #P3932), and cells added at 5.0 × 10^4^ cells/well in assay medium 4 h prior to the experiment.

Analysis of results utilized *XFe Wave Software* (Seahorse Bioscience). Cellular protein levels, determined by using Pierce^tm^ BCA protein assay kit following standard procedure, were obtained for each well, and all data were normalized to protein content. Additional information about assays is available on the Seahorse Bioscience website: http://www.seahorsebio.com

### Cellular ATP measurements

The levels of ATP in CRC cells with altered expression of FASN were assessed using ATP colorimetric assay according to the manufacturer's protocol (#K354-100, BioVision, Milpitas, CA).

### Human tissues analysis

Human CRC and matching normal colonic tissues were obtained from consented patients at the Markey Cancer Center. Tissues collected from the Surgical Pathology Laboratory after surgical resection were immediately processed and then analyzed by western blot analysis and/or IHC.

### Immunohistochemistry

Nineteen clinical specimens of primary CRC with matching normal colonic mucosa and liver metastases were analyzed for expression of FASN (Cell Signaling, # 3180), p62 (Abcam, ab56416) and pAMPK (Cell Signaling, # 2535). Blind scoring performed by a pathologist used a semi-quantitative method. The extent of expression score was assessed on a scale of 0 to 3 (no positive cells = 0, <10% = 1, 10%–50% = 2, positive staining of >50% = 3); the intensity score was also measured on a scale of 0 to 3 (negative = 0, weak = 1, moderate = 2, strong = 3). Multiplication of the values for intensity and extent of expression provided a score for immunoreactivity.

### Statistical analysis

Descriptive statistics and bar graphs summarize the following endpoints: OCR, ECAR, FAO, and ATP levels. Comparisons of non-treated control (NTC) vs. FASNsh groups used a two-sample *t*-test or ANOVA. Statistical analyses were performed using SAS 9.3. Data provided in Figure 2 is an average of area under the curve, and calculated using ANOVA and Tukey post test (the XF Glycolysis Stress Test Software, Seahorse Bioscience). Paired tests using the Wilcoxon signed rank test were employed to assess differential expression of ordinal biomarker immunoreactivity scores between matched normal versus tumor and normal versus metastatic tissues. The correlations between biomarkers were measured using Spearman's correlation coefficient.

## SUPPLEMENTARY FIGURES AND TABLES


