# Supplementary material for: Increased expression of fatty acid synthase provides a survival advantage to colorectal cancer cells via upregulation of cellular respiration
Source: Oncotarget. 2015 Apr 20;6(22):18891–904. doi: 10.18632/oncotarget.3783 (PMC4662462; doi:10.18632/oncotarget.3783)
Supplement: Supplementary file 1 [file oncotarget-06-18891-s001.pdf]

SUPPLEMENTARY FIGURES AND TABLES

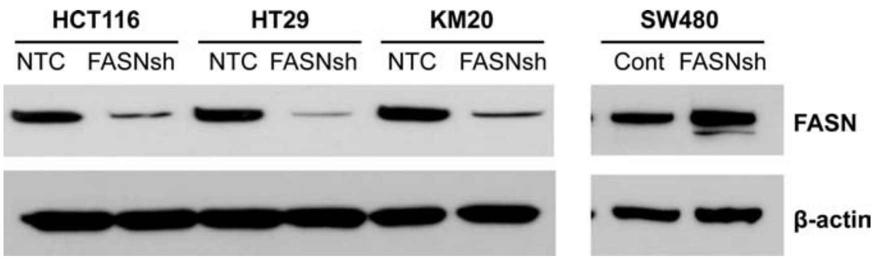

Supplementary Figure S1: Expression of FASN in control and stable knockdown/overexpression CRC cell lines utilized in the study.

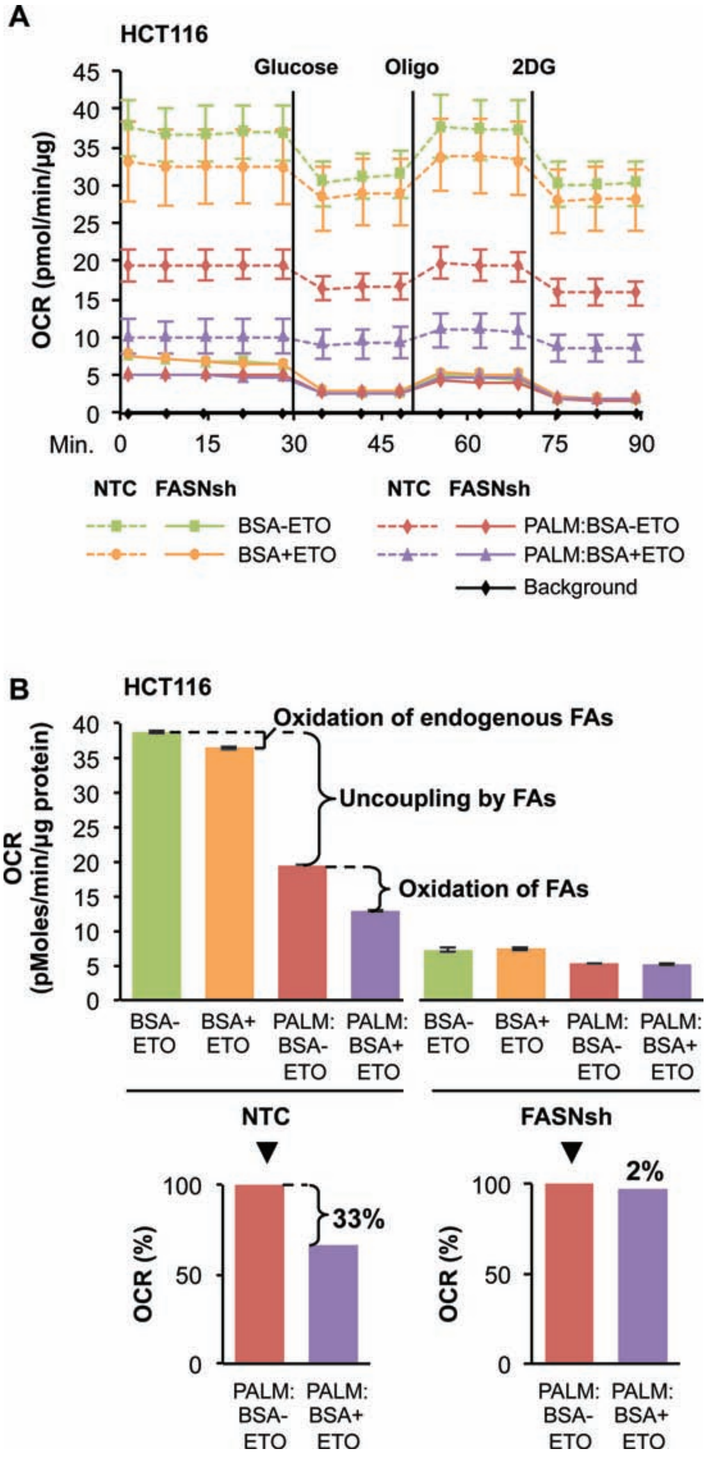

Supplementary Figure S2: Inhibition of FASN decreases OCR and impairs β-oxidation of endogenous fatty acids and exogenous palmitate in HCT116 cells. A. Representative graph of FAO assay using XF Palmitate-BSA FAO substrate (Seahorse Bioscience) in HCT116 cells. The cellular OCR was measured following the exposure to palmitate. Experiment performed according to the protocol provided by Seahorse Bioscience and integrates the XF Cell Mito Stress Test with the BSA control, XF Palmitate reagent and ETO. The oligomycin injection suggests significant uncoupling by palmitate in NTC HCT116 and slight uncoupling in FASNsh HCT116 cells. An increase in maximum respiration beyond the BSA control in NTC HCT116 cells indicates utilization of exogenous FAs. **B.** Quantitative analysis OCR in BSA control and palmitate exposed HCT116 cells. ETO treatment indicates that exogenous fatty acids are primarily utilized for FAO by NTC, but not by FASNsh HCT116 cells.

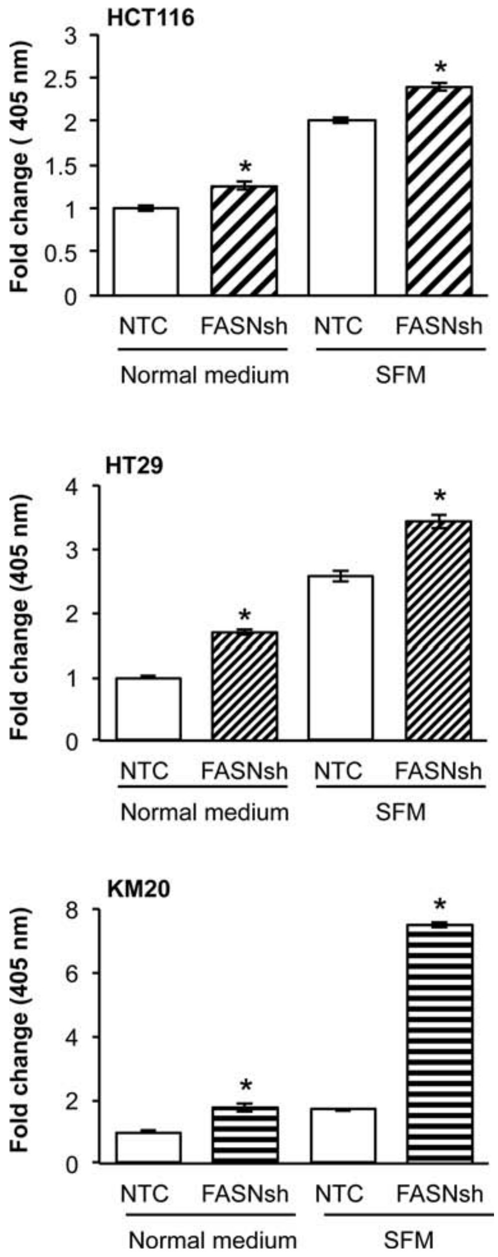

Supplementary Figure S3: Knockdown of FASN induces apoptosis in CRC cells. DNA fragmentation was measured in CRC cell lines with stable knockdown of FASN cultured in normal and SFM conditions for 48 h using Cell Death ELISA (Roche).

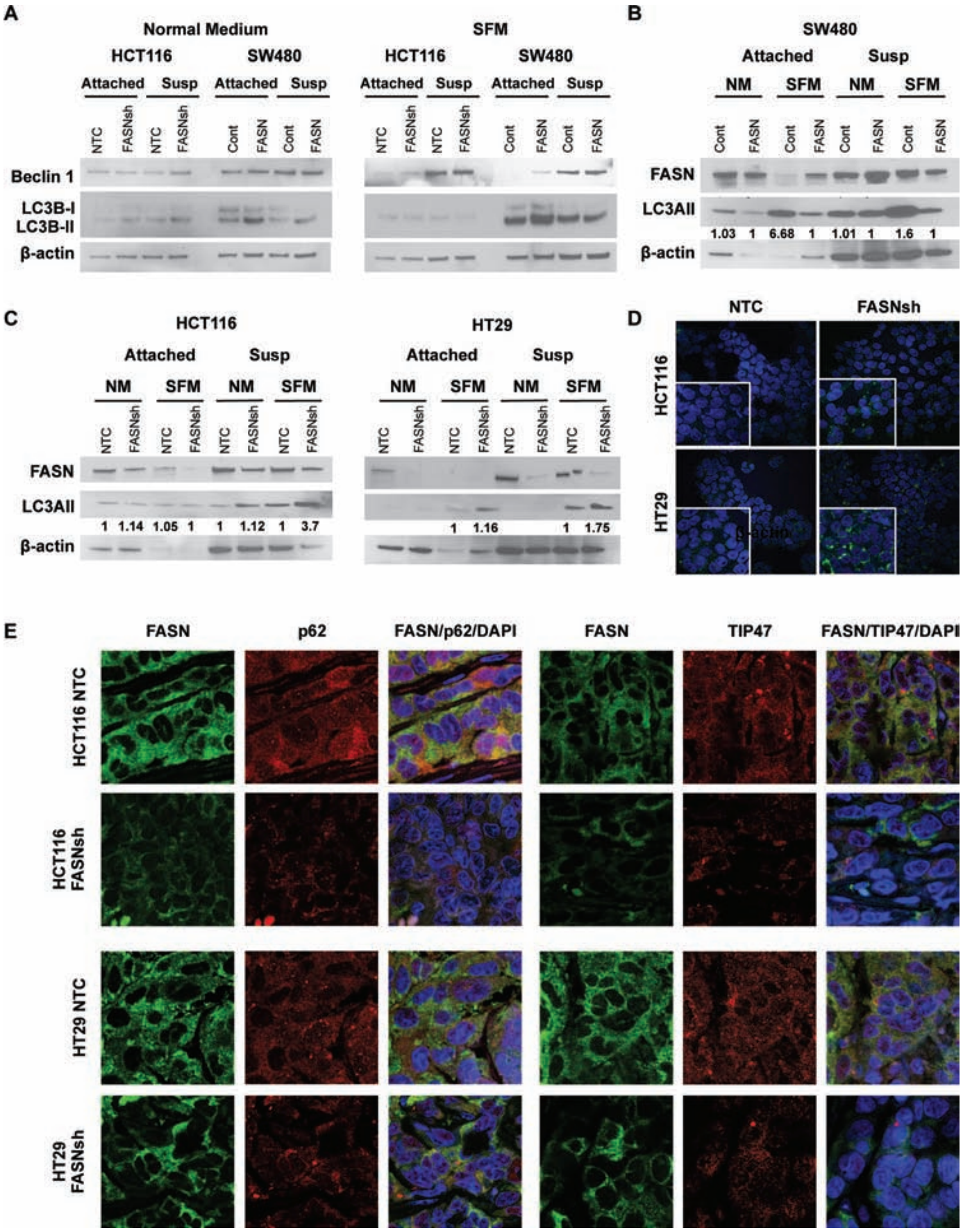

Supplementary Figure S4: Expression of autophagy markers in CRC cells and orthotopic CRCs with altered expression of FASN. A. Western blot analysis of CRC cell lines with altered expression of FASN for Beclin 1 (Cell Signaling, #4122) and LC3B (Sigma, L7543). **B-C.** Western blot analysis of CRC cell lines with altered expression of FASN for LC3A (Cell Signaling, # 4599). The relative expression of LC3A was quantified by normalizing band intensity to actin and is shown under the corresponding bands as a fold change between NTC vs FASNsh or control vs FASN. **D.** Confocal microscopy of HCT116 and HT29 cells with stable knockdown of FASN cultured in SFM for 24 h and stained for LC3A (green) and DAPI (blue). x60 magnification. **E.** Confocal microscopy of immunofluorescent staining of tissue sections from orthotopic CRCs (NTC and FASNsh HCT116; NTC and FASNsh HT29) for FASN (green), p62 (red), TIP47 (red), and DAPI (blue) examined by confocal microscopy. x60_x4 magnification.

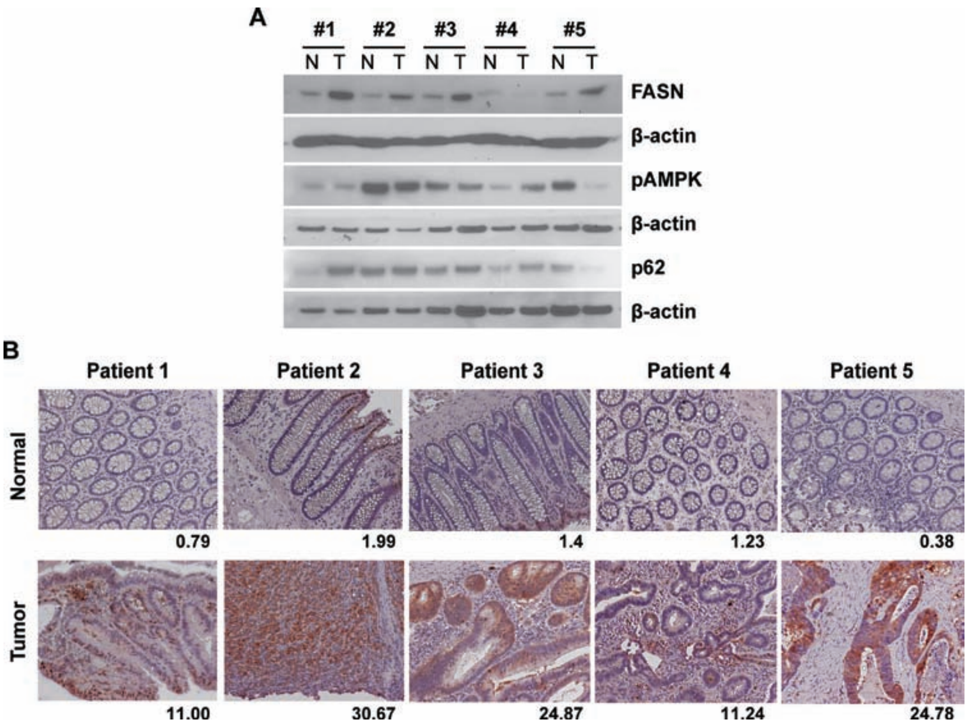

Supplementary Figure S5: A. Western blot analysis of surgical specimens (tumor [T] and matching normal [N] colon) from consented CRC patients (*n* = 5) for expression of FASN, p62 and pAMPK. Specimens were collected and processed immediately after resection. B. IHC staining of tumors and matching normal colons for p62 is shown in the same order as western blot analysis. p62 positive staining was quantified with Nikon Elements Advanced Research image-analysis software and is shown as a percentage of positively stained area.
